# Unmet supportive care needs among cancer patients: exploring cancer entity-specific needs and associated factors

**DOI:** 10.1007/s00432-024-05715-4

**Published:** 2024-04-12

**Authors:** Franziska Springer, Anja Mehnert-Theuerkauf, Claudia Gebhardt, Jens-Uwe Stolzenburg, Susanne Briest

**Affiliations:** 1 Department of Medical Psychology and Medical Sociology, Comprehensive Cancer Center Central Germany (CCCG), University Medical Center Leipzig, Leipzig, Germany; 2 Department of Urology, Comprehensive Cancer Center Central Germany (CCCG), University Medical Center Leipzig, Leipzig, Germany; 3 Department of Gynecology, Comprehensive Cancer Center Central Germany (CCCG), University Medical Center Leipzig, Leipzig, Germany

**Keywords:** Cancer care, Unmet supportive care needs, Cancer entity, Care needs

## Abstract

**Purpose:**

Recognizing unmet care needs among cancer patients is crucial for improving a person-centered and tailored approach to survivorship care. This study aimed to explore the prevalence of unmet supportive care needs, pinpointing entity-specific areas of burden, and to identify factors associated with unmet needs within a diverse sample of cancer patients.

**Methods:**

In this cross-sectional sub-study of a large multicenter study, 944 adult cancer patients reported supportive care needs via the well-validated SCNS. Most frequent diagnoses included breast (n = 276), prostate (n = 237), hematological (n = 90) and gynecological cancer (n = 74), which were analyzed for entity-specific care needs.

**Results:**

Across most cancer entities, health system and information, and psychological needs were most commonly reported, with fear of the cancer spreading and information regarding cancer control/diminishment ranking as the most prevalent individual concerns. Notable differences in entity-specific needs emerged for gynecological cancer patients, who exhibited more psychological (*p* = 0.007, OR = 2.01) and physical needs (*p* = 0.005, OR = 2.02), and prostate cancer patients, who showed higher sexuality needs (*p* < 0.001, OR = 2.95) but fewer psychological (*p* < 0.001, OR = 0.55), physical (*p* < 0.001, OR = 0.31) and patient care needs (*p* = 0.006, OR = 0.62). Non-distressed participants had fewer supportive care needs in each domain (all *p* < 0.001). Patients with functional impairments and female respondents reported increased unmet needs across most domains.

**Conclusion:**

The high prevalence of patients feeling inadequately informed about their disease and care aspects, particularly among those with functional impairments, reflects a key challenge in the healthcare system. Specific interventions and improvements in patient-doctor communication are essential to address cancer entity-specific care needs.

**Supplementary Information:**

The online version contains supplementary material available at 10.1007/s00432-024-05715-4.

## Introduction

A cancer diagnosis and its associated treatments pose multifaceted challenges to patients across various dimensions of life, encompassing physical, psychological, social, spiritual, and economic burdens (Broemer et al. 2021; Carrera et al. 2018; Lee et al. 2015; Visser et al. 2010). The primary aim of comprehensive supportive cancer care is to provide personalized support, addressing the specific needs of patients across health promotion and prevention, active cancer treatment phases, cancer survivorship, and palliation (Fitch 2008). Due to advancements in cancer diagnostic and treatment, the population of cancer survivors is growing, thus representing a challenge for the health care system (Atun and Cavalli 2018). In an aging society with multiple and chronic diseases it is becoming even more demanding to provide tailored and effective support to each patient.

To enhance both patient outcomes in survivorship care, as well as healthcare system outcomes such as costs, a person-centered approach is essential with regard to offering adequate and effective support tailored to the unique circumstances of the patients (Epstein and Street 2007). Recognizing that unmet supportive care needs are linked to reduced quality of life, anxiety and depression in cancer patients (Cochrane et al. 2022; Paterson et al. 2023), it becomes crucial to address these needs through targeted interventions, such as professional support services or clinical trials. Unmet supportive care needs refer to areas of burden subjectively reported by patients, indicating a desire for additional support or information beyond what is currently provided. The initial step towards optimizing tailored supportive cancer care, meeting the needs of patients as best as possible and to improve satisfaction with care involves identifying the most prevalent areas of burden (Maguire et al. 2015).

Numerous systematic reviews have explored unmet supportive care needs within various tumor populations, such as breast (Fiszer et al. 2014), lung (Cochrane et al. 2022), gynecological (Beesley et al. 2018; Maguire et al. 2015), colon (Kotronoulas et al. 2017), prostate (Prashar et al. 2022), hematological (Tsatsou et al. 2021), and mixed cancer cohorts (Lisy et al. 2019). A recent umbrella systematic review (Paterson et al. 2023), encompassing 30 systematic reviews and synthesizing data from 612 publications, highlighted shared patterns across different cancer types. The most frequent unmet supportive care needs, as revealed by this review, consistently encompassed psychological and emotional needs, as well as a lack of information concerning the healthcare system, e.g. understanding the diagnosis, symptoms, and treatments. A substantial proportion of patients, ranging from 40 to 90%, report at least one unmet need (Abu-Odah et al. 2022; Boyes et al. 2012; Lam et al. 2011), with fear of cancer recurrence being the most common issue detected in many studies. However, distinctive stressors inherent to each cancer type also lead to individual needs. Notably, breast cancer patients tend to report more unmet needs compared to those with prostate or colorectal cancer (Li et al. 2013; Moreno et al. 2019), while gynecological and prostate cancer patients exhibit a predominant concern with sexuality and intimacy issues (Beesley et al., 2018; Cockle-Hearne et al. 2013; Maguire et al. 2015; Watson et al. 2016). Thus, generalizability of previous findings is compromised due to variations across study results, and a predominant focus on specific cancer types, such as breast or lung cancer.

In addition to the type of cancer, several psychological, medical and sociodemographic factors have been associated with an increased risk for unmet supportive care needs, such as advanced cancer, symptom burden, younger age, mental health issues, social support and income (Beesley et al. 2018; Boyes et al. 2012; Fiszer et al. 2014; Lam et al. 2011; Sarkar et al. 2015; Tsatsou et al. 2021). Identifying factors associated with specific supportive care needs might help to identify patients who are at high-risk of not having their needs met and may therefore experience adverse psychosocial and physical consequences.

The primary aim of this study, therefore, is to investigate the prevalence of unmet supportive care needs within a large cohort of cancer patients across all entities. Secondly, we aim to evaluate cancer entity-specific care needs, and thirdly, to identify further disease-related and sociodemographic factors that are associated with unmet supportive care needs.

## Methods

### Study design and participants

Adult cancer patients included in this cross-sectional sub-study were recruited as part of a larger German multicenter study from diverse healthcare settings, including acute care hospitals, outpatient cancer care facilities, and cancer rehabilitation clinics. For the present study, only the data obtained from patients at the study center Hamburg are being analyzed, as the measures of interest in supportive care needs were only assessed within this particular subsample.

Inclusion criteria encompassed a confirmed malignant tumor diagnosis, age between 18 and 75, and being fluent in German language. Exclusion criteria considered severe physical, cognitive, or verbal impairments hindering the ability to provide informed consent. Ethics committee approval was obtained (Hamburg file number: 2768), and the study protocol has been published (Mehnert et al. 2012). Written informed consent was obtained from all participants before study participation.

Eligible patients were invited to complete a battery of validated self‐report measures. Additionally, a subset of participants underwent a structured clinical interview, the findings of which are presented elsewhere (Mehnert et al. 2014). Data collection took place from July 2008 to November 2010.

### Measures

Sociodemographic information (age, gender, partnership, income, employment) was gathered via standardized self-report questionnaires. Medical characteristics (tumor entity, date of diagnosis, UICC disease stage, metastases, treatment intention, cancer treatments, functional impairment (Karnofsky index)) were collected through medical records.

The *Supportive Care Need Survey* (SCNS) was developed specifically for the oncological context and assesses adult cancer patients’ perceived needs as a result of having cancer (Boyes et al. 2009; Lehmann et al. 2012). The 34-item instrument comprises five domains: *psychological*, *health system and information*, *physical and daily living*, *patient care and support*, and *sexuality* needs. The *psychological* domain encompasses various aspects, including anxiety, feeling down or depressed, fears about the cancer spreading or uncertainty about the future. Within the *health system and information* domain, patients may report a need to be given written information about important aspects of care, information about aspects of managing the illness and side effects at home, being treated like a person not just another case, or being informed about cancer which is under control or diminished. The *patient care and support* domain involves the need for more choice about which hospital to attend or which specialists to see, and hospital staff attending promptly to physical needs. *Physical and daily living* needs address issues such as pain, lack of energy or work around the home. Lastly, *sexuality needs* encompass changes in sexual feelings or sexual relationships (for more details of each domain see Table S1). The scale assesses issues that patients experienced, the needs that remain unmet and the magnitude of these needs on a five-point Likert scale (1 = no need, not applicable; 2 = no need, satisfied; 3 = low need; 4 = moderate need; 5 = high need). For each domain a patient is categorized as having “no to low” need, if their score for every item in a domain was 1, 2 or 3. Conversely, patients are categorized as having a “moderate to high” level of need, if they indicate a need of 4 or 5 for at least one item in the respective domain. This ensures that only patients with unmet cancer-related needs are identified as such. The scale shows high internal consistency for the five domains, with Cronbach’s α ranging from 0.86 to 0.96 and its validity was confirmed (Boyes et al. 2009).

Psychological distress was assessed with the one-item German *Distress Thermometer* (DT) (Mehnert et al. 2006) on a visual analog scale from 0 (no distress) to 10 (extreme distress). A value ≥ 5 has been indicated in the literature as a cut-off for a clinically relevant level of distress (Mehnert et al. 2018).

### Statistical analysis

Sample characteristics of sociodemographic and medical characteristics were displayed descriptively.

Frequencies of supportive care needs were presented for the entire sample, that is the proportion of patients reporting each supportive care need domain, as well as the most frequently reported issue on an item level. The sample was then stratified according to psychological distress (DT < 5 vs. ≥ 5) and differences in each need domain were analyzed using chi-square-tests. Secondly, cancer entity-specific needs were analyzed accordingly for the entire sample, however, only in the most frequent cancer entities due to statistical power (breast, prostate, hematological and gynecological cancer). In order to identify entity-specific need domains, chi-square-tests were run for each entity (e.g. breast cancer vs. all others).

We explored factors that are associated with each supportive care need domain through logistic regression models. Multivariate logistic regression models were applied separately to each domain and were adjusted for all relevant factors identified in univariate logistic regression models (*p* < 0.05). The sociodemographic and medical characteristics investigated encompassed age, gender, partnership, income, treatment intention, time since diagnosis, UICC disease stage, presence of metastases, functional impairment, and the type of cancer treatments received.

Effect sizes for all chi-square-tests and logistic regression models were reported as odds ratio (OR) that may range from 0 to infinite. All statistical tests were conducted as two-tailed tests with an alpha level set at 5%. The analyses were performed using the latest version of R statistical software (R Core Team 2021).

## Results

Out of 1450 eligible patients, 1,016 were included in the study (response rate of 70.1%). In total, 944 patients completed the questionnaire and were considered for our analyses. Patients included in our analyses (n = 944) were younger than non-responders (n = 506) (57.7 vs. 60.5 years, *p* < 0.001) and more likely to have a curative treatment intention (70.5% vs. 55.5%, *p* = 0.01). No difference in gender was observed (*p* = 0.11).

The mean age of participants was 57.7 years, with 52.2% being female (Table 1). The most frequent cancer types were breast (29.2%) and prostate cancer (25.1%). The majority of the patients (70.5%) indicated a curative treatment intention.

**Table 1:** Sociodemographic and medical sample characteristics (n = 944)

	n	%
Sociodemographic characteristics		
Age in years, Mean (SD, range)	57.7 (12.0, 18–75)	
Gender, female	493	52.2
Marital status		
Married	617	67.4
Single	138	15.1
Divorced	108	11.8
Widowed	52	5.7
Partner, yes	686	77.7
Occupation		
Employed	419	45.6
Retired^a^	390	42.4
Other	110	12.0
Income per month < 2000€	538	66.5
Medical characteristics		
Tumor site		
Breast	276	29.2
Prostate	237	25.1
Hematological	90	9.5
Gynecological^b^	74	7.8
Colon/rectum	57	6.0
Lung	46	4.9
Head and neck	29	3.1
Stomach/esophagus	22	2.3
Kidney/urinary tract	16	1.7
Malignant melanoma	14	1.5
Other	83	8.8
Months since current diagnosis, Mean, Median (SD)	13.4, 6 (18.6)	
Treatment intention		
Curative	666	70.5
Palliative	195	20.7
Currently not assessable	83	8.8
Currently receiving psychosocial support	112	12.0

n is based on valid answers and therefore sometimes does not add up to the full sample

^a^Including retirement and disability pension

^b^Mostly cervix (n = 28), ovarian (n = 17) and uterine cancer (n = 17)

### Supportive care needs

**Fig. 1:**
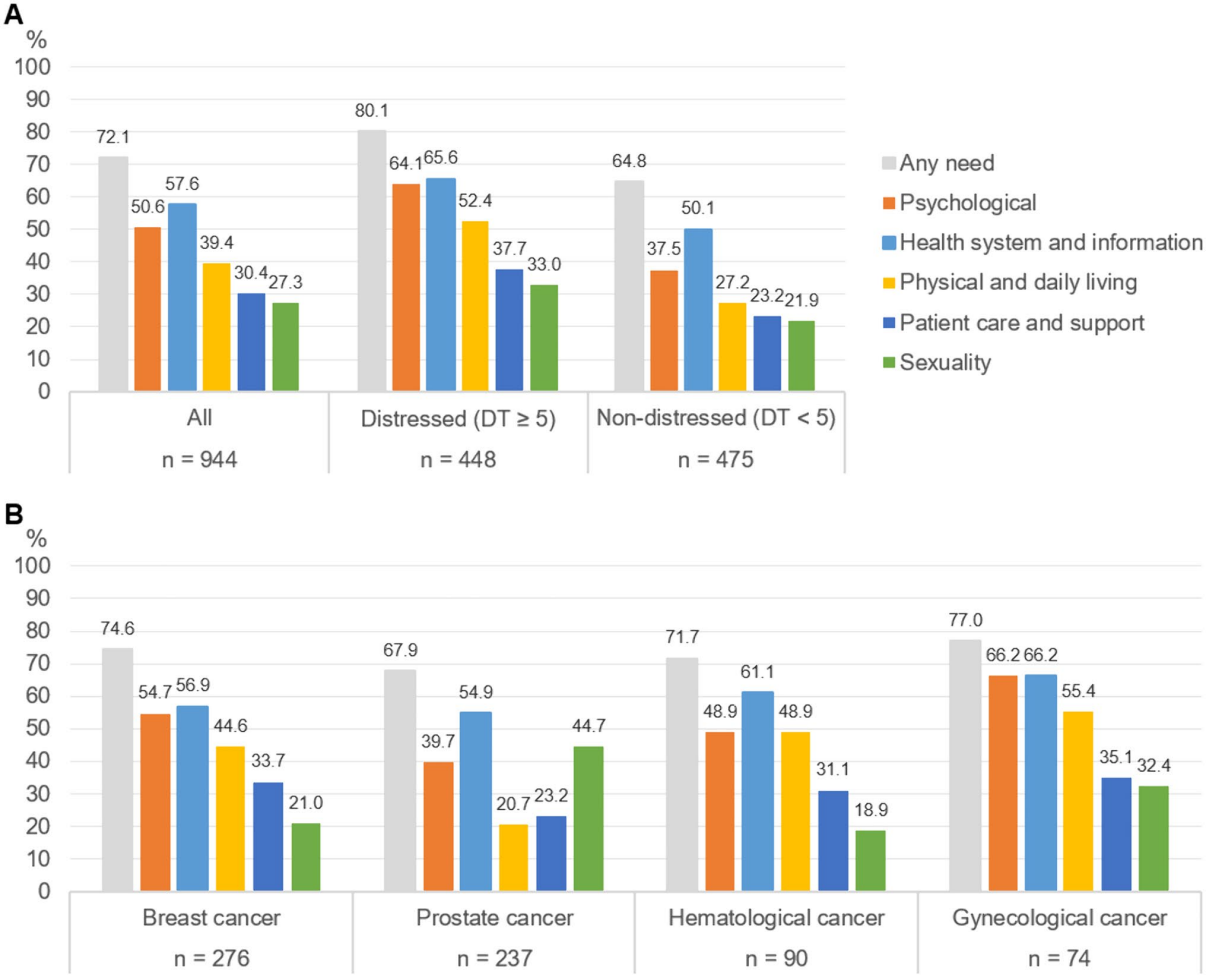
Supportive care needs according to the SCNS domains. *SCNS* Supportive Care Need Survey, *DT* Distress Thermometer. **A** Supportive care need domains in the entire sample and stratified by psychological distress (DT < 5 vs. ≥ 5). **B** Supportive care need domains stratified by cancer entity

The most frequent unmet supportive care needs were related to *health system and information*, mentioned by 57.6% (n = 544) of the patients, followed by *psychological* needs (50.6%, n = 478) (Fig. 1A). Additionally, a substantial proportion of patients reported unmet needs in the domains of *physical and daily living* (39.4%, n = 372), *patient care and support* (30.4%, n = 287), and *sexuality* issues (27.3%, n = 258). In total, 72.1% (n = 681) of the sample reported at least one unmet need across all domains. Stratifying the sample based on psychological distress (DT < 5 vs. ≥ 5) leads to a consistent pattern of supportive care needs in both groups (Fig. 1A). However, non-distressed patients exhibited fewer supportive care needs in each domain (all *p* < 0.001, OR(psychological) = 0.34, OR(health system) = 0.53, OR(physical) = 0.34, OR(patient care) = 0.50, OR(sexuality) = 0.57).

On an item level, the most frequently reported issues included the fear of the cancer spreading (38.5%, n = 339) and the need for information regarding the cancer which is under control or diminishing (i.e. remission) (40.2%, n = 345).

### Cancer entity-specific needs

When examining different cancer entities (Fig. 1B), *health system and information* needs, along with *psychological* needs, consistently emerged as the most frequently reported domains, with the exception of prostate cancer patients. The latter reported a predominant unmet need related to *sexuality* issues in addition to *health system and information* needs.

For breast and gynecological cancer patients, the most prevalent issue was the fear of the cancer spreading (breast: 45.6%, gynecological: 54.3%). Hematological cancer patients most frequently expressed the need for information about the cancer which is under control or diminishing (43.6%). In contrast, prostate cancer patients reported issues such as the desire for information about sexual relationships (37.3%) and being informed about cancer which is under control or diminishing (38.4%).

Notable differences emerged in the need domains differentiated by cancer entities. Breast cancer patients reported more *physical* (*p* = 0.04, OR = 1.35) but fewer *sexuality* needs (*p* = 0.007, OR = 0.62) compared to other cancer patients. Gynecological cancer patients indicated more *psychological* (*p* = 0.007, OR = 2.01) and *physical* needs (*p* = 0.005, OR = 2.02). Prostate cancer patients reported higher *sexuality* needs (*p* < 0.001, OR = 2.95), but fewer *psychological* (*p* < 0.001, OR = 0.55), *physical* (*p* < 0.001, OR = 0.31) and *patient care* needs (*p* = 0.006, OR = 0.62). No differences were observed in hematological cancer patients.

### Associated factors with supportive care needs

When controlling for relevant factors identified in univariate regression models, *psychological* and *patient care* needs were more prevalent in women and patients with higher functional impairment (Table 2). *Health system and information* needs were more frequently reported by patients with higher functional impairment. *Physical* needs were more frequently expressed by women, patients with higher functional impairment, and those receiving chemotherapy. Conversely, *sexuality* needs were more frequently reported by men, patients with a partner and those under curative treatment intention. Factors such as time since cancer diagnosis, disease stage, metastases and cancer treatments other than chemotherapy showed no significant impact on the level of supportive care needs.

**Table 2:** Associated factors with supportive care needs (SCNS)

	Univariate regression model			Multivariate regression model		
	b	OR [CI 95%]	*p*	b	OR [CI 95%]	*p*
Psychological needs						
Age	− 0.03	0.97 [0.96, 0.98]	** < 0.001**	− 0.02	0.98 [0.97, 0.99]	** < 0.001**
Gender^a^	− 0.69	0.50 [0.39, 0.65]	** < 0.001**	− 0.52	0.60 [0.45, 0.79]	** < 0.001**
Functional impairment^b^	− 0.02	0.98 [0.97, 0.99]	**0.005**	− 0.01	0.99 [0.98, 0.99]	**0.047**
Chemotherapy	0.43	1.54 [1.19, 1.99]	**0.001**	0.11	1.11 [0.83, 1.49]	0.47
Health system and information needs						
Age	− 0.01	0.99 [0.98, 0.99]	**0.02**	− 0.01	0.99 [0.98, 1.00]	0.14
Gender	− 0.31	0.73 [0,56, 0.95]	**0.02**	− 0.22	0.80 [0.61, 1.06]	0.12
Functional impairment	− 0.02	0.98 [0.97, 0.99]	** < 0.001**	− 0.02	0.98 [0.97, 0.99]	** < 0.001**
Physical and daily living needs						
Age	− 0.03	0.97 [0.96, 0.99]	** < 0.001**	− 0.01	0.99 [0.98, 1.01]	0.34
Gender	− 0.85	0.43 [0.33, 0.56]	** < 0.001**	− 0.78	0.46 [0.32, 0.65]	** < 0.001**
Partner	− 0.47	0.62 [0.45, 0.86]	**0.004**	− 0.15	0.86 [0.60, 1.25]	0.43
Income^c^	− 0.60	0.55 [0.40, 0.74]	** < 0.001**	− 0.14	0.87 [0.60, 1.25]	0.45
Functional impairment	− 0.04	0.96 [0.95, 0.98]	** < 0.001**	− 0.04	0.96 [0.95, 0.98]	** < 0.001**
Chemotherapy	0.65	1.92 [1.47, 2.51]	** < 0.001**	0.34	1.40 [1.01, 1.96]	**0.046**
Patient care and support needs						
Age	− 0.02	0.98 [0.97, 0.99]	** < 0.001**	− 0.01	0.99 [0.98, 1.01]	0.22
Gender	− 0.76	0.47 [0.35, 0.62]	** < 0.001**	− 0.77	0.46 [0.32, 0.67]	** < 0.001**
Partner	− 0.44	0.65 [0.46, 0.90]	**0.01**	− 0.09	0.91 [0.63, 1.34]	0.64
Income	− 0.39	0.68 [0.49, 0.93]	**0.02**	− 0.03	0.97 [0.66, 1.41]	0.86
Functional impairment	− 0.02	0.98 [0.96, 0.99]	** < 0.001**	− 0.02	0.98 [0.96, 0.99]	** < 0.001**
Chemotherapy	0.29	1.33 [1.01, 1.76]	**0.046**	− 0.04	0.96 [0.68, 1.35]	0.82
Sexuality needs						
Gender	0.53	1.70 [1.27, 2.27]	** < 0.001**	0.53	1.70 [1.22, 2.37]	**0.002**
Partner	0.67	1.96 [1.33, 2.95]	** < 0.001**	0.49	1.62 [1.08, 2.51]	**0.02**
Treatment intention^d^	− 0.52	0.59 [0.40, 0.86]	**0.01**	− 0.51	0.60 [0.39, 0.91]	**0.02**
Chemotherapy	− 0.47	0.62 [0.46, 0.84]	**0.002**	− 0.08	0.93 [0.65, 1.31]	0.67

Significant values (p < 0.05) are marked in bold

Displayed are all significant factors (p < 0.05) from univariate regression models for each supportive care need domain. These factors are then entered in the respective multivariate regression models

*SCNS* Supportive Care Need Survey, *OR* odds ratio, *CI* confidence interval

^a^Binary, reference category female

^b^KARNOFSKY index, higher values represent lower functional impairment

^c^Binary, reference category < 2000€ per month

^d^Binary, reference category curative treatment intention

## Discussion

In this cross-sectional study, three out of four cancer patients reported at least one unmet supportive care need. Entity-specific areas of burden could be identified for some tumor types. In general, we could confirm results of previous research, namely the overall high level of cancer patients with unmet supportive care needs (Andreu et al. 2022; Boyes et al. 2012; Fiszer et al. 2014; Paterson et al. 2023).

Our results underline a consistent pattern of identified needs across various cancer types, as well as in distressed and non-distressed patients, aligning well with numerous previous studies (e.g. Andreu et al. 2022; Beesley et al. 2018; Boyes et al. 2012; Fiszer et al. 2014; Hart et al. 2022; Kotronoulas et al. 2017; Lam et al. 2011; Moreno et al. 2019; Paterson et al. 2023; Tsatsou et al. 2021). These needs predominantly revolve around challenges of navigating in the healthcare system, in particular a lack of information concerning cancer diagnosis and treatments, alongside psychological concerns. In order to improve planning of supportive care for patients with unmet needs, screening for distress has been demonstrated to be useful (Springer et al. 2023). However, the overall high prevalence of unmet care needs implies that access to existing information resources and support services might be obstructed for patients in need. Nevertheless, providing information to address unmet needs does not always completely alleviate uncertainties inherent in cancer trajectories, and certain needs may persist despite informational support. The aim of supportive care might therefore be to mitigate unmet needs and uncertainties with adequate information and support services to a manageable degree rather than eradicating them entirely. Achieving this requires providing high-quality and easily comprehensible information resources that need to be flexibly delivered, encompassing both traditional paper as well as digital formats. Digital information resources and support show promising effects on patients’ distress, anxiety and depression (Springer et al. 2024). The delivery of support should be seamlessly integrated into the inpatient and outpatient care settings, provided by healthcare professionals at the right moments throughout the cancer trajectory.

In addition, our results outline that one in two patients indicates unmet psychological needs, including the fear of cancer relapse, uncertainty about the future, anxiety or depression. This underlines the importance of routine screening for psychological distress within clinical cancer care and the provision of psycho-oncological support. Given its high frequency, also demonstrated in previous studies (Abu-Odah et al. 2022; Beesley et al. 2008, 2018; Boyes et al. 2012; Fiszer et al. 2014; Kotronoulas et al. 2017; Lam et al. 2011; Lisy et al. 2019; Moreno et al. 2019), future research should increasingly focus on the fear of cancer recurrence (FCR) or progression (FoP), investigate its prevalence, enhance the identification of at-risk patients, and contribute to developing accessible written and verbal psychoeducational resources. While a systematic review demonstrated the effectiveness of various psychological interventions on improving FCR outcomes (Tauber et al. 2019), the authors assert that there is still limited understanding of the most efficacious treatment components for alleviating FCR symptomatology. To address this gap, tailored interventions specifically targeting FCR/FoP need to be developed, validated, and made accessible.

We have demonstrated cancer entity-specific care needs in diverse need domains. Breast cancer patients exhibit elevated physical needs, aligning well with previous studies (Li et al. 2013; Moreno et al. 2019). This might be attributed to specific breast cancer treatment consequences, e.g. after mastectomy, that may result in body image concerns and pain. Mastectomy rates are increasing, especially in patients younger than 50 years (Kummerow et al. 2015; Trocchi et al. 2019). A large study involving more than 1.2 million early-stage breast cancer patients, revealing that 36% of them underwent mastectomy (Kummerow et al. 2015). The increase of mastectomy was seen in patients with node negative breast cancer and in situ disease, probably due to better possibilities of breast reconstruction. However, a considerable decrease of the mastectomy rate in women who underwent mammography screening was observed in a large German study (Trocchi et al. 2019). Breast cancer patients may thus prioritize addressing physical problems before discussing sexuality-related issues. Further analyses in specific subgroups and with different treatment procedures are needed. The elevated prevalence of psychological and physical care needs in gynecological cancer patients, observed in our study, aligns well with previous research (Beesley et al. 2018; Maguire et al. 2015; Steele and Fitch 2008). Unmet needs in gynecological patients may stem from the type of cancer itself, affecting reproductive organs. This, in turn, could potentially influence the women’s sense of identity, femininity and sexuality. Additionally, treatment procedures such as surgery or radiation may result in changed body functionality and physical appearance, leading to elevated levels of distress and support needs. However, in contrast to previous studies, we did not observe an increased risk concerning sexuality needs in gynecological cancer patients. This may be attributed to the comparison group, which primarily comprised prostate cancer patients exhibiting even higher levels of sexuality needs.

Conversely, overall low care needs in prostate cancer patients may be attributed to gender differences in stigma and communication, as well as an early diagnosis and often slow progression of the disease compared to other cancers. Nevertheless, it is noteworthy that these patients exhibit a distinct and alarming high prevalence of sexuality issues (Bond et al. 2019; Cockle-Hearne et al. 2013; Moreno et al. 2019; Prashar et al. 2022; Watson et al. 2016) that remain unmet by the healthcare system. This may be directly linked to long-term effects of cancer treatment, such as the common side effect of sexual or erectile dysfunction, and should be targeted with tailored interventions and psychosocial support offers.

Functional impairment seems to be a pivotal risk factor for unmet needs (Armes et al. 2009; Beesley et al. 2018) and becomes a growing challenge for the healthcare system, especially in light of an aging population and an increase of multiple comorbid and chronic diseases in elderly patients. It is further associated with longer hospital stays and worse survival (Lage et al. 2020). Dealing with a decrease in functionality due to comorbidities requires a high level of health literacy and self-management. Supporting these patients with their needs becomes a complex and demanding, yet necessary task. Rehabilitation thereby represents a key component of supportive care (Stucki 2021).

In addition, undergoing a chemotherapy increased multifaceted needs in our results, encompassing physical, psychological and patient care needs, as also supported by previous studies (Andreu et al. 2022; Beesley et al. 2008; O’Brien et al. 2017). Physical issues may directly stem from the treatments’ toxicity, while psychological concerns may be related to feelings of hopelessness and a more pessimistic outlook on the future in the case of chemotherapy. However, caution is warranted in interpreting these findings, as, when controlled for other relevant factors, chemotherapy remained significant only for physical needs. Sexuality needs, on the contrary, mainly evident in male and prostate cancer patients (Armes et al. 2009; Bond et al. 2019), reveal distinct associated factors. These can be directly linked to their impact on intimate relationships. Specifically, having a partner increasing the need for support, and a curative treatment intention may offer an outlook on the cancer survivorship phase, fostering a desire to return to a normal life, including sexual relationships.

### Clinical implications

The considerable number of patients expressing not feeling properly informed about their disease and aspects of their care reflects the current challenge within the healthcare system. Particularly, patients with pronounced functional impairments or comorbid medical conditions, and those confronted with complex treatment or health-related information, require assistance in managing their diseases and treatment regimens. Improvements in communication between patients and healthcare providers, along with multidisciplinary collaboration in both inpatient and outpatient settings, may help to improve clinical care processes and to address unmet supportive care needs.

Given the potential constraints imposed by available personal and financial resources in the healthcare system, a strategic approach is essential to optimize psychosocial support planning and delivery. Distressed patients thereby may be targeted first, emphasizing the importance of implementing a routine distress screening procedure as an integral component of standard clinical cancer care. Furthermore, a targeted focus on patients with heightened risk, such as women and those undergoing chemotherapy, is reasonable. Specific cancer departments may tailor their screening protocol and support offerings to the unique needs prevailing within their entity-specific domain. For instance, urological cancer departments may incorporate routine screening and support addressing sexuality issues and intimate relationships.

### Strengths and limitations

This study examined a large and diverse cohort of cancer patients, encompassing various tumor types and treatment intentions, with a balanced gender distribution. This heterogeneity enhances the generalizability of our findings and enables comparisons between different tumor entities. Validated medical data extracted from medical charts were available. However, cancer entity-specific analyses were only possible for certain tumor types, due to partly small sample sizes. Future research should investigate distinct care needs in rare cancer types to draw robust conclusions and advance personalized support. Notably, our sample of hematological and gynecological cancer patients was relatively small, and results should thus be interpreted with caution. In addition, study participants were younger and exhibited a greater proportion of curative treatment intention compared to non-responders. Results should be interpreted with caution and may not be applicable for older patients with cancer and with palliative treatment intention. Additionally, it is noteworthy that the distribution of items across different care need domains is uneven, potentially introducing bias towards domains with a greater number of items, i.e. health system and information and psychological needs. However, it is important to recognize that during the validation of the scale, items that were identified as most useful in the clinical setting were carefully selected from a broader pool of potential items (Boyes et al. 2009). This validation process thus underscores the significance of specific domains within the healthcare setting. As highlighted by previous studies (Abu-Odah et al. 2022; Paterson et al. 2023), culture-specific aspects of supportive care needs may come into play and our data cannot be generalized to non-Westernized, low- to medium-income countries. Cultural variations in healthcare systems, cancer care delivery and programs may impact the prevalence of care needs that remain unmet.

## Conclusion

A significant proportion of cancer patients reports unmet supportive care needs across diverse need domains. Adapting cancer care to address entity-specific problems seems crucial, and particularly gynecological cancer patients exhibit a higher number of unmet needs. In contrast, prostate cancer patients generally report fewer needs, except for a notable emphasis on sexuality issues. Our findings offer valuable insight for healthcare providers on optimizing care planning with available resources. In addition, they underline the growing challenge faced by the healthcare system in delivering personalized care to patients dealing with functional impairment within multiple and complex treatment regimens.

## Supplementary Information

Below is the link to the electronic supplementary material.

Supplementary file1 (DOCX 16 KB)

## Data Availability

The datasets generated during and/or analyzed during the current study are available from the corresponding author upon reasonable request.
