# Supplementary material for: Unmet supportive care needs among cancer patients: exploring cancer entity-specific needs and associated factors
Source: J Cancer Res Clin Oncol. 2024 Apr 12;150(4):190. doi: 10.1007/s00432-024-05715-4 (PMC11009727; doi:10.1007/s00432-024-05715-4)
Supplement: Supplementary file 1 — Supplementary file1 (DOCX 16 KB) [file 432_2024_5715_MOESM1_ESM.docx]

**Supplementary file**

| **Table S1**. Need domains of the Supportive Care Needs Survey (SCNS-34) |
| --- |
| **Health system and information** |
| 1. Being informed about your test results as soon as feasible |
| 2. Being informed about cancer which is under control or diminishing (that is, in remission) |
| 3. Being given explanations of those tests for which you would like explanations |
| 4. Being adequately informed about the benefits and side effects of treatments before you choose to have them |
| 5. Having one member of hospital staff with whom you can talk to about all aspects of your condition, treatment and follow up |
| 6. Being treated in a hospital or clinic that is as physically pleasant as possible |
| 7. Being given information (written, diagrams, drawings) about aspects of managing your illness and side-effects at home |
| 8. Being informed about things you can do to help yourself to get well |
| 9. Being treated like a person not just another case |
| 10. Being given written information about the important aspects of your care |
| 11. Having access to professional counselor (e.g. psychologist, social worker, counselor, nurse specialist) if you, your family or friends need it |
| **Psychological** |
| 1. Uncertainty about the future |
| 2. Anxiety |
| 3. Feeling of sadness |
| 4. Fears about the cancer spreading |
| 5. Feeling down and depressed |
| 6. Learning to feel in control of your situation |
| 7. Worry that the results of treatment are beyond your control |
| 8. Keeping a positive outlook |
| 9. Feelings about death and dying |
| 10. Concerns about the worries of those close to you |
| **Physical and daily living** |
| 1. Not being able to do the things you used to do |
| 2. Work around the home |
| 3. Pain |
| 4. Feeling unwell a lot of the time |
| 5. Lack of energy/tiredness |
| **Patient care and support** |
| 1. More choice about which cancer specialists you see |
| 2. More choice about which hospital you attend |
| 3. Reassurance by medical staff that the way you feel is normal |
| 4. Hospital staff acknowledging, and showing sensitivity to, your feelings and emotional needs |
| 5. Hospital staff attending promptly to your physical needs |
| **Sexuality** |
| 1. To be given information about sexual relationships |
| 2. Changes in sexual feelings |
| 3. Changes in your sexual relationships |
